# Hydration, Eating Attitudes and Behaviors in Age and Weight-Restricted Youth American Football Players

**DOI:** 10.3390/nu13082565

**Published:** 2021-07-27

**Authors:** Susan Yeargin, Toni M. Torres-McGehee, Dawn Emerson, Jessica Koller, John Dickinson

**Affiliations:** 1 Exercise Science Department, Arnold School of Public Health University of South Carolina, Columbia, SC 29208, USA; torresmc@mailbox.sc.edu; 2 Department of Physical Therapy, Rehabilitation Sciences, and Athletic Training, School of Health Professions University of Kansas Medical Center, Kansas City, KS 66160, USA; demerson@kumc.edu; 3 Surgi-Care Inc., Boston, MA 02451, USA; jkoller328@gmail.com; 4 Palmetto Health/USC Orthopedic Center, Columbia, SC 29203, USA; johndickinson333@yahoo.com

**Keywords:** sweat rate, fluid consumed, pathogenic eating behaviors, hypohydration, American tackle football

## Abstract

There is a paucity of research examining hydration and nutrition behaviors in youth American football players. A potentially unique risk factor are league restrictions based on weight (WR) or age (AR). The purpose of this study was to examine hydration status between WR and AR leagues. The secondary purpose was to describe eating patterns in players. An observational cohort design with 63 youth football players (10 ± 1 yrs, 148.2 ± 9.4 cm, 44.9 ± 15.3 kg) was utilized. Independent variables were league (AR (*n* = 36); WR (*n* = 27)) and activity type (practice (PX = 8); game (GM = 3)). Dependent variables were hydration status (urine osmolality; percent change in body mass (%BM)), eating attitudes (Children’s Eating Attitude Test (ChEAT-26)) and self-reported frequency of meals. On average, players arrived activity mildly hypohydrated (830 ± 296 mOsm/kg) and %BM was minimal (−0.1 ± 0.7%) during events. Players consumed 2 ± 1 meals and 1 ± 1 snack before events. The ChEAT-26 survey reported 21.6% (*n* = 8) of players were at risk for abnormal eating attitudes. Among these players, eating binges, vomiting, excessive exercise and drastic weight loss were reported. Youth American football players arrived activity mildly hypohydrated and consumed enough fluid during activity to maintain euhydration. Abnormal eating attitudes and the use of unhealthy weight loss methods were reported by some youth American football players.

## 1. Introduction

Pop Warner is the largest youth American football organization in the world and greater than 250,000 children participate per season. Pop Warner football, in an attempt to make the sport safer, sometimes divide their players by age and weight [1]. To ensure these rules are followed, players weigh-in at the beginning of the season and before each game to confirm they are not over the weight limit for that specific team [1]. If they weigh-in at a different weight, then the child may not be allowed to play that day or eventually have to change teams. Leagues without weight restrictions divide their players into teams by age only. However, if there is a larger child on a team (usually greater than 36.3 kg), playing restrictions are typically enforced for that child to ensure safety and encourage fair play.

The National Health and Nutrition Examination Survey, estimated 15.7% of boys 2–19 years old in the US are overweight, 19.1% obese and 6.3% severely obese [2]. When examining boys closer to youth football age range, US boys aged 6–11 years old revealed 20.4% obese [2]. The health risks posed by hypohydration and improper nutrition are heightened for athletes participating in sports that involve weight restrictions. The pressure of these restrictions may lead to unhealthy weight loss methods, which negatively affect the overall function of the body and may predispose athletes for exertional heat illness (EHI) [3,4]. As mentioned previously, youth weight and age play a major role in the structure of the team composition in youth football. If a youth athlete is overweight, or is in the early maturation stage, it is implied their larger size may put them at higher risk for injury or potentially injuring other athletes. This not only puts an early stigma that being overweight or obese increases higher risk for injury, but may also put a young athlete at higher risk to engage in compensatory behaviors to induce weight loss (e.g., excessive exercise, fasting, self-induced vomiting, use of laxatives, diuretics, appetite suppressants, etc.). These behaviors align with previous research that revealed 12% of adolescent athletes used one or more compensatory behaviors to control weight [5].

When working with youth athletes with weight pressures, it is important to recognize hypohydration and improper diet are predisposing factors of EHI [6,7,8]. Hypohydration can be intentional when athletes engage in activities that induce passive or active dehydration; using a sauna or exercising in sweat suits were found to be the most common in adolescent athletes [5]. Additionally, poor diet is considered a risk factor for EHI, as low electrolyte levels alter normal muscle/organ function and low energy leads to early muscle fatigue during physical activity [3,9,10]. When examining adolescent male wrestlers and the influence of dietary restriction; a reduction in protein and muscular performance was evident, but there was little effect on linear growth and maturation [11]. There is a robust association between abnormal eating attitudes and behaviors (i.e., disordered eating, eating disorders (ED), pathogenic behaviors) and their contribution to children being overweight and obese [12]. It is important to recognize any association between dietary restraints, dieting and ED risk in the context of pediatric weight management, particularly as the onset of ED risk peaks during adolescence [13]. This early recognition will help in the prevention of unhealthy weight loss methods in this young population.

Youth and adolescent males participating in American football compose the majority of EHI cases that present to United States emergency rooms [14]. Research reveals youth football has the highest EHI rate compared to high school and collegiate teams [15]. Risk factors for EHI in the American football population include uniforms, high body mass index (BMI), environmental conditions, poor physical condition, lack of heat acclimatization and improper work to rest ratio [3,6,7,9]. Beginning physical activity and maintaining a euhydrated state is crucial to support efficient thermoregulation and cardiovascular function [3,9,10]. Studies have concluded high school athletes are hypohydrated before the start of activity and underestimate sweat losses and fluid needs [16,17]. This has been noted in youth football players at summer camp as well [18]. It is unknown if this is true for youth football players during a regular season.

There is a substantial amount of research regarding hypohydration in adult athletics and the effects of unhealthy weight loss methods in weight-restricted sports [11,19,20,21,22,23,24,25,26,27,28]. However, there is a scarcity of research in children and in football leagues who utilize weight restricted (WR) or age restricted (AR) guidelines. Therefore, the primary purpose of our study was to examine hydration and eating frequency (number of meals and snacks per/day) among youth football leagues (WR vs. AR) during different activities (practice: PX vs. games: GM). The secondary purpose was to determine the prevalence of ED risk by assessing eating attitudes and compensatory behaviors. We hypothesized that the WR league would have greater hypohydration than the AR league. We also hypothesized ED risk and compensatory behaviors would exist in youth football players.

## 2. Materials and Methods

### 2.1. Design

We utilized a cross-sectional research design. The independent variables were football league (age (AR) and weight (WR)) and activity type (practice (PX) or game (GM)). The dependent variables were hydration status (urine osmolality (Uosm), percent change in body mass (%BM)), fluid consumed (FC), sweat rate (SR), eating attitudes (Children’s Eating Attitude Test (ChEAT-26)) and self-reported eating frequency.

### 2.2. Participants

Youth football players (*n* = 63) between the ages of 8–13 years from local recreational leagues in the southeastern region of the United States participated. The convenience sample was recruited during the team parent pre-season meeting. The WR league included participants from 4 teams of specific weight and age categories. Each player within the WR league had to be within the weight range before the season began to be eligible for that division. Additionally, at each game, participants had to weigh in, with full pads and meet requirements to play that day. Players within the AR league were assigned teams by age only. There were no exclusions to participate in the study. The study was approved by the University of South Carolina’s Institutional Review Board (Pro00024799) and written assent and informed consent were obtained from participants and their parent/legal guardian prior to baseline testing.

### 2.3. Instrumentation and Measurements

Hydration Status: A Multi-Sample Osmometer (Advanced Instrument model 2020) was calibrated before each set of urine samples and determined player’s pre activity Uosm [29,30]. Samples were run in duplicate. If the values were more than 15 mOsm/kg apart, a triplicate sample was analyzed. The two closest samples were averaged to represent the sample. Each athlete’s weight was obtained in kilograms (Tanita TBF 300A, Tokyo, Japan) while wearing only shorts. Percent change in body mass (%BM) was calculated as: [(pre event kg–post event kg)/pre event kg] × 100 [3].

Fluid Consumed and Sweat Rate: Each player was provided a 1 L bottle and could fill it with their normal self-provided drink of choice. FC was tracked throughout PX and GM with fluids added if desired by the player. At the end of activity, the remaining fluid was measured to the nearest 10 mL mark on a graduated cylinder. Sweat rate (SR) was calculated as: [(pre event kg–post event kg) + L of FC)]/hours of exercise [3].

Eating Disorder Attitudes and Compensatory Behaviors: Children’s Eating Attitude Test (ChEAT-26) is a modified version of the Eating Attitudes Test (EAT-26) for children between 7 and 14 years old [31,32,33]. The ChEAT-26 is composed of 26 questions with subscales related to dieting, restricting, purging and food preoccupation and 5 questions related to compensatory behaviors (e.g., binging-eating more than you would normally eat, self-induced vomiting-made yourself throw-up, use of diet pills, excessive exercise, or lost a significant amount of weight in a short period of time). Participants rated their responses using a 6-point Likert scale ranging from 1 to 6 (i.e., never, rarely, sometimes, often, very often, always). A higher score is representative of more problematic or maladaptive eating attitudes and a cut-off score of 20 is indicative of risk for ED pathology. To be considered at risk for ED, we used two criteria: (1) participants scored greater than 20 on the ChEAT-26 and/or (2) participants met the criteria for compensatory behaviors or recent significant weight loss. This instrument does not diagnose an ED, but only aids in identifying characteristics of an ED [33,34,35]. The ChEAT-26 has an internal consistency of 0.79 and for this study was 0.80 [36].

Self-Reported Eating Frequency: Participants were asked to self-report their eating frequency prior to each observed PX and GM. We measured eating frequency by asking how many meals and snacks were eaten prior to activity (PX and GM) the same day in whole numbers.

### 2.4. Procedures

An informational meeting explained the study to parents and players before consent/assent was obtained. Basic demographics were collected at this time (age, height, weight). The ChEAT-26 was given to participants to complete after the first PX, but prior to the first GM. Data collection occurred on 7 (AR) or 8 (WR) nonconsecutive practices and the first 3 games of the season for both leagues. The WR had two practices on the last observed day (9:00 am and 6:00 pm). For the most part, practices for both leagues commonly occurred at 6:00 pm. Start times for WR games ranged from 10:00 am–1:00 pm whereas the AR leagues games started at 7:00 pm. Details regarding work to rest ratios of PX and GM have been described previously [37]. When participants arrived at activity (PX and GM) they self-reported their meal/snack frequency and were then given a collection cup to provide a urine sample. Players were weighed before and after activity. There was no intervention by the researchers during PX or GM. Players participated in football as normal and asked to follow their normal hydration behaviors.

### 2.5. Data Analysis

Statistical Analysis Software (SAS Institute Inc., Cary, NC, USA) was used for hydration behavior variables; and we used SPSS Statistical Software (Version 27; SPSS Inc., Armonk, NY, USA) and alpha < 0.05 for eating behavior variables. G*Power software (version 3.1.9.2., Heinrich Heine University, Dusseldorf, Germany) was used to calculate power for ED risk [38]. Using an alpha of 0.05 and a large effect size (0.7), our power calculation indicated we needed a sample of 34 participants, with estimated power being 0.90. Descriptive statistics were calculated for all dependent and descriptive variables. Differences in Uosm, %BM, FC and SR least square means were assessed between leagues (AR, WR) and activities (PX, GM) with ANOVA or general linear model procedures depending on if the data was complete and balanced, or incomplete and unbalanced, respectively. Frequencies and proportions with 95% confidence intervals were calculated for categorical variables (e.g., ED risk, binge eating, purging, use of diet pills, diuretics and laxatives, excessive exercise and drastic weight loss) along with means and standard deviations for continuous variables (e.g., total ChEAT-26 score and dieting, restricting and purging and food preoccupation subscale scores).

## 3. Results

A total of 63 youth football players participated in the study. The WR (*n* = 27, 10 ± 1 years old, 144 ± 9 cm, 37.6 ± 8.0 kg) and AR (*n* = 36, 11 ± 1 years old, 152 ± 8 cm, 50.1 ± 17.1 kg) players included all positions and levels of experience. Limited data were available for ChEAT-26 (*n* = 37), whereas 63 participants completed the remaining measures but data were missing at some time points.

### 3.1. Demographics

Participant demographics are presented in Table 1. Athletes in the WR and AR youth football leagues were within the age range of 9–13 years old, but there was a statistically significant difference in age between leagues (*p* = 0.003). Although the participants were close in age, the AR players were taller (*p* = 0.001) and weighed more (*p* < 0.001) than WR.

### 3.2. Hydration Status

Descriptive statistics for Uosm and %BM are presented in Table 2. Uosm ranged from 78 to 1383 mOsm/kg pre activity. There was no interaction between league and activity type (F = 1.35, *p* = 0.240) for Uosm. The AR league had higher Uosm than the WR league (F = 6.01, *p* = 0.015), but there was no main effect for activity type (F = 0.52, *p* = 0.471). Percent body mass change within an activity session ranged from −2.4% to +2.0%. There was a significant interaction between league and activity type for %BM (F = 5.44, *p* = 0.020) with WR having a greater %BM after games. A main effect existed for league (F = 4.51, *p* = 0.034), but not activity type (F = 0.18, *p* = 0.666).

### 3.3. Fluid Consumed and Sweat Rate

Descriptive statistics for FC are presented in Table 3. FC between GM and PX was not different (*p* = 0.272). There was a statistically significant difference in FC between WR and AR during activity (PX and GM) (*p* = 0.045). There was no difference in FC between WR and AR during GM (*p* = 0.072) or during PX (*p* = 0.378). The WR players consumed less fluid during GM than PX (*p* = 0.048). There was no difference in FC when comparing GM and PX for AR (*p* = 0.929). There was a positive correlation between FC and SR (r = 0.875).

Descriptive statistics for SR are presented in Table 3. The maximum SR value recorded in a player was 1.3 L/hr during a PX. There was a significant difference in SR between GM and PX (*p* = 0.013). AR had a greater SR during activity (PX and GM) than WR players (*p* = 0.028). There was also a statistically significant difference in SR when comparing GM and PX for WR (*p* = 0.004). There was no difference in SR when comparing GM and PX for AR (*p* = 0.311).

### 3.4. Eating Disorder Attitudes and Compensatory Behaviors

Only 37 youth football players completed the ChEAT-26. The estimated prevalence for ED risk for all youth football players was 21.6% (*n* = 8); of those, 5.4% (*n* = 2) were at risk based on the ChEAT-26, 13.5% (*n* = 5) on compensatory behaviors and 2.7% (*n* = 1) from both the ChEAT-26 and compensatory behaviors. WR league accounted for 87.5% (*n* = 7) of ED risk and AR for 12.5% (*n* = 1). Overall, 16.2% (*n* = 6) of our participants reported use of compensatory behaviors, with 10.8% (*n* = 4) reporting binge eating where they felt that they may not be able to stop, at least 2–3 times per month; 2.7% (*n* = 1) had vomited to control their weight or shape at least once a month, 5.4% (*n* = 2) had exercised more than 60 min a day to lose or to control their weight at least once a day and 2.7% (*n* = 1) noticed they lost a significant amount of weight in the last 6 months. None of our participants reported use of laxatives, diet pills or diuretics to control their weight. Overall, the player’s self-reported current weight and their ideal weight only differed by 1.2 kg as 25% (*n* = 9) indicated their current weight was their ideal weight. The remaining players indicated both a lower ideal weight (29.7%, *n* = 11) and a greater ideal weight (43.2%, *n* = 16).

### 3.5. Self-Reported Eating Frequency

Overall, youth football players had an average of 2 ± 1 meals and 1 ± 1 snack prior to activity (PX and GM). This also represents the average eating frequency prior to PX. Before GM, players overall self-reported 1 ± 1 meal and 1 ± 1 snack with WR reporting 1 ± 0 and AR reporting 2 ± 1. There were players who reported 0 meals and snacks prior to PX (*n* = 2) and GM (*n* = 4). There were no statistical differences for meals and snacks between leagues (*p* > 0.05)

## 4. Discussion

The purpose of our study was to examine hydration status and eating frequency in WR and AR youth American football leagues during PX and GM. We found players from both leagues were mildly hypohydrated. We hypothesized that the WR league would have greater hypohydration than the AR league. Contrary to our hypothesis, we found AR players experienced greater hypohydration compared to WR. Our data support our hypotheses that youth football players exhibit ED risk and compensatory behaviors, finding approximately 20% of players self-reported items that indicated ED risk on the ChEAT or compensatory behaviors. Eating frequency seemed more dependent on event type.

### 4.1. Hydration Status

Urine osmolality indicated that our participants arrived to activity (PX and GM) hypohydrated, with the exception of WR pre PX. According to generally accepted guidelines [29], participants’ hypohydration was mild on average. There were individual players with Uosm values that could be described as severe hypohydration at PX and GM (maximum values recorded ranged from 1128–1383 mOsm/kg) [29]. Similar pre-activity hypohydration has been found in youth athletes participating in soccer and other sports [17,39,40]. Specifically in youth football, up to 70% of players arrived to camp and morning practices hypohydrated with individual variances [18,41]. Our study confirms this, as on average 64% of our players arrived at least mildly hypohydrated for a PX or GM.

AR players were more hypohydrated than WR players, particularly before PX. Data collection for WR PX started before school began in August, while school had started when AR began PX in September. The WR players were likely coming to PX from home, while AR may have been coming to PX straight from school or after-school care. This may explain why AR had higher Uosm values, as they may not have had a chance to hydrate sufficiently during school before PX. WR players had higher Uosm values before their GM than PX (not statistically). One explanation may be the start time for WR games, which began at 10:00 am and players may have had less time between waking and the GM to consume food and fluids. It is also possible WR players may have purposely not consumed as much water if they were concerned about passing the weigh-in prior to the game.

Our participants had small changes in %BM during activity (PX and GM). This indicates our participants maintained their pre hydration status throughout activity by matching sweat losses with FC. Our results, coupled with previous research in young athletes [17,18,39,41], solidifies a common theme: dehydration during activity is minimized when fluids are accessible, but athletes do not optimize the time between activity sessions to reach euhydration.

### 4.2. Fluid Consumed and Sweat Rate

The average FC of our participants was similar to studies with adolescent soccer, volleyball and football players and youth campers [16,17,18,41,42]. Specifically, previous research in youth football campers participating in three sessions per day recorded an average of 0.76 L of fluid per hour during activity [18]. Campers consumed enough fluid to account for sweat loss during activity [18]. Similarly, high school players consumed fluids equivalent to two-thirds of their sweat loss [16,18]. Our participants, both AR and WR players, consumed enough fluids to make up for their sweat losses during activity. Fluid consumed increased as sweat rate increased as noted by the positive correlation. However, the strong relationship can be partly explained by FC calculated within the SR equation.

Sweat rates in our study were likely affected by a variety of factors including hydration status of the players, intensity of activity and environmental conditions [10]. Age-restricted players were larger than WR; therefore, AR players may have had greater increases in metabolic heat generation resulting in higher SR. However, though there was statistical difference in SR between leagues, any clinical significance in relation to anthropometrics would need to be determined in future research. Practice SR was greater than GM in our results, which is in contrast to McDermott et al. (2009) that reported a larger SR during GM than PX. Differences may exist because of the timing of each activity was different in both studies. SR is a valuable variable to calculate in athletes to determine fluid needs. If players drink the amount of fluid that they are losing through sweat per hour, then hydration status can be maintained.

### 4.3. Eating Disorder Characteristics and Behaviors

Our study is the first to examine ED risk in American youth tackle football players (boys) who participated in either a WR or AR league. We found approximately 20% of players were at higher risk for ED attitudes and compensatory behaviors compared to previous research that estimated 14.4% of youth male athletes (from various sports) were at risk for Eds [5,43,44,45,46,47,48]. Additionally, it has been noted in previous research that pediatric EDs are more common than type 2 diabetes and the higher rates of EDs are evident in younger children, boys and minority groups [49,50,51]. However, generally speaking, male athletes typically have lower scores than their female counterparts, as well as their non-athlete controls [43]. In our study, regardless of WR or AR league, we also found low ChEAT-26 scores in our youth football players. Of those who were, the majority were in the WR league. Youth football players who were at risk for an ED may find themselves in a vulnerable developmental period in which they are faced with a range of general as well as sport-specific risk factors. For example, adolescence is characterized by growth, physical changes and personality development [5]; and/or adolescent athletes’ frequent drive to “be their best” combined with performance pressures from coaches and/or parents can create an overly competitive environment contributing to increase risk for ED attitudes and use of compensatory behaviors [44].

Boys involved in sports that necessitate weight restrictions include not only wrestlers, but also youth football players, gymnast, runners, swimmers, etc. In these sports, “cutting weight” may provide an edge to qualify for competition, reduce restrictions for competition, or to enhance performance. Vomiting, eating binges and excessive exercise to manipulate weight was self-reported by a small number of youth football players in our study. These behaviors have also been observed in high school wrestlers [23,25]. In approximately 2500 high school wrestlers during a single season, most took part in at least one unhealthy weight loss method per week [23]. Findings across multiple youth sport revealed ~ 8% of youth athletes reported they were trying to lose weight and 12% reported use of one or more compensatory behaviors; with the most common behaviors included activities inducing passive or active dehydration (e.g., sauna, exercise in sweat suite, etc.) [5]. Our study revealed ~16% reported use of compensatory behaviors, with the most common compensatory behaviors being binge eating and excessive exercise. Our participants who reported use of unhealthy weight loss methods may have been utilizing these methods to lose weight for football. Unhealthy weight loss methods cause hypohydration and electrolyte imbalances, which alters normal thermoregulatory system and organ function. In combination with exercise, this can lead to EHI and other health related risks [4,6,7]. Unhealthy compensatory behaviors in sport may commonly be perceived as a method to “cut weight”; however, these behaviors may also be used to “gain weight”. Sports, such as football, may unintentionally portray the message that “bigger is better” to enhance performance for certain positions. Thus, in turn, possibly putting pressures on athletes to binge eat to gain weight. Future research should examine if pressures related to eating are position specific. Our results indicated that some youth football players wanted a lower ideal weight, while others wanted a greater weight.

### 4.4. Self-Reported Eating Frequency

Intensive physical training and participation in competitive sports during childhood/adolescence may affect athletes’ pubertal development. Contrarily, maturational timing, early or late, may impact the athletes’ development and the selection of their sport and/or sport position. Therefore, it is important to assure that youth athletes are appropriately fueling. Although our study did not examine daily dietary intake and actual number of consumed, calories, we identified the frequency of meals and snacks per day. We reported general differences in self-reported eating behaviors between PX and GM. This may have been due to the time-of-day PX and GM took place. Practices started at 6 pm for both leagues and GM for AR were at 7 pm. However, GM started at 10 am, 11:30 am and 1 pm for WR; therefore, WR players would not have eaten as many meals and snacks as they would have eaten before PX. Food intake findings could have also been influenced by WR players purposely not eating as much on game days because of the weigh-in prior to the GM. Similar to WR youth football, wrestling also has weight restrictions and limiting food consumption is sometimes used by wrestlers as a weight control method to ensure participation [23]. Studies have shown that during high school wrestling season, there is a decrease in protein consumption, body protein and fat stores in wrestlers [26]. In addition, wrestlers’ weight and muscular strength and power significantly increase during the post season when compared to mid-season [20,26]. Not eating enough nutrients prior to activity can decrease energy levels and performance and it is a predisposing factor for EHI [4,7,52]. It is recommended parents and coaches familiarize themselves with the energy/nutritional requirements (incorporating total energy expenditure, plus exercise energy expenditure/physical activity levels and energy deposits of growing tissues) for youth populations which have been previously published [53]. This will provide parents and coaches a better understanding not only of the fueling needs of youth athletes but how to plan the frequency of meals when schedules do not align with standard mealtimes.

### 4.5. Strengths and Limitations

We are the first study to examine hydration status of American tackle football players younger than high school during a regular practice and competition season. We are also the first to examine eating attitudes in this population. A limitation included that PX and GM started at different times during the day, confining the comparisons of hydration status and eating behaviors of players between PX and GM. Environmental conditions were different on each day of data collection which has the potential to impact sweat rates. Variables were self-reported data and questions could have been answered untruthfully. There was also inconsistency with participation due to the young age of the players, parents not getting their children to PX and GM early enough and participants playing at different field locations at the same time resulting in some datasets being incomplete or unbalanced. These reasons also explain the low numbers of players completing the ChEAT questionnaire. Future research should focus on evaluating hydration and nutrition knowledge of youth football parents and coaches. It would also be beneficial to repeat this study with youth football players for an entire season. Examining specifically what types of food youth football players are eating and reasons for their eating behaviors would provide additional insight into the patterns presented in our study.

## 5. Conclusions

Overall, youth football players arrived to PX and GM slightly hypohydrated, although players in the AR league were more hypohydrated than WR. It seems that the players understood the importance of hydrating during physical activity and consumed enough fluids during PX and GM to match SR. Meal and snack frequency were adequate for practices but could be considered limited for game days. As we hypothesized, we observed the existence of ED risk and compensatory eating behaviors in this group of youth football players. This was noted in both leagues, but particularly in the WR league. This may be a result of players trying to alter their weight for football. The incorporation of hydration guidelines (similar to wrestling) and nutrition education for athletes and their parents should be considered for youth football leagues to help prevent the use of unhealthy weight loss methods.

## Figures and Tables

**Table 1:** Descriptive statistics of all participants (*n* = 63) and by league (WR: *n* = 27 and AR: *n* = 36).

	Aggregated			WR			AR			
	M(SD)	Min	Max	M(SD)	Min	Max	M(SD)	Min	Max	*p*-Value
Age (y)	11(1)	9	13	10(1)	9	12	11(1) ^a^	9	13	0.003
Height (cm)	148(9)	127	167	144(9)	127	163	152(8) ^a^	133	167	0.001
Weight (kg)	44.7(15.2)	26.2	95.7	37.6(8.0)	26.2	56.3	50.1(17.1) ^a^	27.7	95.7	<0.001

All values are represented in mean (SD) and minimum and maximum values. WR: weight-restricted league; AR: age-restricted league. ^a^: AR > WR.

**Table 2:** Descriptive statistics for pre activity urine osmolality and percent change in body mass.

	Agg			WR			AR		
	Agg	PX	GM	Agg	PX	GM	Agg	PX	GM
Uosm (mOsm/kg)									
Mean (SD)	829 (296)	823 (307)	855 (251)	786 (291)	770 (312)	839 (200)	899 (290) ^a^	903 (282)	886 (330)
Min–Max	78–1383	78–1383	184–1262	78–1321	78–1321	184–1262	196–1383	287–1383	196–1215
%BM									
Mean (SD)	+0.1 (0.7)	+0.1 (0.6)	+0.1 (0.9)	+0.1 (0.7)	+0.2 (0.7)	0.0 (1.0) ^b^	+0.2 (0.6)	+0.1 (0.6)	+0.4 (0.6)
Min–Max	+2.4–−2.0	+2.4–−2.0	+1.5–−1.8	+2.4–−2.0	+2.4–−2.0	+1.5–−1.8	+2.1–−1.4	+2.1–−1.4	+1.2–−0.9

Agg: Aggregated; WR: Weight-Restricted League; AR: Age-Restricted League; Uosm: pre activity urine osmolality; %BM: percent change in body mass after activity; ^a^: AR > WR; ^b^: WR > AR.

**Table 3:** Sweat rate and fluid consumed of all youth football players (*n* = 63) from weight (*n* = 27) and age (*n* = 36) restricted leagues, during combined practices and games.

	Agg			WR			AR		
(L/hr)	Agg	PX	GM	Agg	PX	GM	Agg	PX	GM
SR	0.4 (0.2)	0.5 (0.2) ^b^	0.4 (0.2)	0.4 (0.2)	0.4 (0.2)	0.4 (0.2)	0.5 (0.2) ^a^	0.5 (0.2)	0.4 (0.2)
FC	0.4 (0.2)	0.4 (0.2)	0.3 (0.2)	0.4 (0.2)	0.4 (0.2) ^b^	0.3 (0.2)	0.4 (0.2)	0.4 (0.2)	0.4 (0.2)

All values are represented in mean (SD). Agg: Aggregated; WR: Weight-Restricted League; AR: Age-Restricted League; ^a^: AR > WR; ^b^: PX > GM.

## Data Availability

The data presented in this study are openly available in openICPSR at [https://www.doi.org/], accessed on 25 July 2021, reference number openicpsr-146124.
